# Molecular epidemiology of *Mycobacterium tuberculosis* in Brazil before the whole genome sequencing era: a literature review

**DOI:** 10.1590/0074-02760200517

**Published:** 2021-03-15

**Authors:** Emilyn Costa Conceição, Richard Steiner Salvato, Karen Machado Gomes, Arthur Emil dos Santos Guimarães, Marília Lima da Conceição, Ricardo José de Paula Souza e Guimarães, Abhinav Sharma, Ismari Perini Furlaneto, Regina Bones Barcellos, Valdes Roberto Bollela, Lívia Maria Pala Anselmo, Maria Carolina Sisco, Cristina Viana Niero, Lucilaine Ferrazoli, Guislaine Refrégier, Maria Cristina da Silva Lourenço, Harrison Magdinier Gomes, Artemir Coelho de Brito, Marcos Catanho, Rafael Silva Duarte, Philip Noel Suffys, Karla Valéria Batista Lima

**Affiliations:** 1 Fundação Oswaldo Cruz-Fiocruz, Instituto Nacional de Infectologia Evandro Chagas, Programa de Pós-Graduação em Pesquisa Clínica e Doenças Infecciosas, Rio de Janeiro, RJ, Brasil; 2 Fundação Oswaldo Cruz-Fiocruz, Instituto Nacional de Infectologia Evandro Chagas, Laboratório de Bacteriologia e Bioensaios em Micobactérias, Rio de Janeiro, RJ, Brasil; 3 Fundação Oswaldo Cruz-Fiocruz, Instituto Oswaldo Cruz, Laboratório de Biologia Molecular Aplicada a Micobactérias, Rio de Janeiro, RJ, Brasil; 4 Universidade Federal do Rio Grande do Sul, Programa de Pós-Graduação em Biologia Celular e Molecular, Porto Alegre, RS, Brasil; 5 Secretaria Estadual de Saúde do Rio Grande do Sul, Centro Estadual de Vigilância em Saúde, Centro de Desenvolvimento Científico e Tecnológico, Porto Alegre, RS, Brasil; 6 Fundação Oswaldo Cruz-Fiocruz, Escola Nacional de Saúde Pública Sergio Arouca, Centro de Referência Professor Hélio Fraga, Laboratório de Referência Nacional para Tuberculose e outras Micobacterioses, Rio de Janeiro, RJ, Brasil; 7 Universidade do Estado do Pará, Instituto de Ciências Biológicas e da Saúde, Pós-Graduação Biologia Parasitária na Amazônia, Belém, PA, Brasil; 8 Instituto Evandro Chagas, Seção de Bacteriologia e Micologia, Ananindeua, PA, Brasil; 9 Instituto Evandro Chagas, Laboratório de Geoprocessamento, Ananindeua, PA, Brasil; 10 International Institute of Information Technology, Department of Data Science, Bangalore, India; 11 Universidade de São Paulo, Departamento de Clínica Médica da Faculdade de Medicina de Ribeirão Preto, Ribeirão Preto, SP, Brasil; 12 Universidade Federal do Rio de Janeiro, Instituto de Microbiologia Paulo de Góes, Laboratório de Micobactérias, Rio de Janeiro, RJ, Brasil; 13 Universidade Federal de São Paulo, Departamento de Microbiologia, Imunologia e Parasitologia, São Paulo, SP, Brasil; 14 Instituto Adolfo Lutz, Centro de Bacteriologia, Núcleo de Tuberculose e Micobacterioses, São Paulo, SP, Brasil; 15 Universit e Paris-Saclay, Ecologie Systematique Evolution, Centre National de la Recherche Scientifique, AgroParisTech, Orsay, France; 16 Coordenação Geral de Vigilância das Doenças de Transmissão Respiratória de Condições Crônicas, Brasília, DF, Brasil; 17 Fundação Oswaldo Cruz-Fiocruz, Instituto Oswaldo Cruz, Laboratório de Genética Molecular de Microrganismos, Rio de Janeiro, RJ, Brasil

**Keywords:** tuberculosis, Mycobacterium tuberculosis, genotyping, MIRU-VNTR typing, RFLP-IS*6110*, Brazil

## Abstract

Molecular-typing can help in unraveling epidemiological scenarios and improvement for disease control strategies. A literature review of *Mycobacterium tuberculosis* transmission in Brazil through genotyping on 56 studies published from 1996-2019 was performed. The clustering rate for mycobacterial interspersed repetitive units - variable tandem repeats (MIRU-VNTR) of 1,613 isolates were: 73%, 33% and 28% based on 12, 15 and 24-loci, respectively; while for RFLP-IS*6110* were: 84% among prison population in Rio de Janeiro, 69% among multidrug-resistant isolates in Rio Grande do Sul, and 56.2% in general population in São Paulo. These findings could improve tuberculosis (TB) surveillance and set up a solid basis to build a database of *Mycobacterium* genomes.

Despite being an ancient disease, tuberculosis (TB) is still the leading cause of death among infectious diseases worldwide. From 2016 to 2020, Brazil has been on the World Health Organization (WHO) list of high burden countries for TB and TB/HIV co-infection.^1^ In 2014, WHO proposed the End TB Strategy that targets TB prevention, care, control, and together with the Sustainable Development Goals (SDGs) aimed at trying to bring TB incidence and mortality on a global level to those observed in high-income countries.^2^ ^,^ ^3^ ^,^ ^4^ ^,^ ^5^ ^,^ ^6^

The three pillars of the End TB Strategy are: (i) integrated, patient-centered TB care and prevention, bold policies, and supportive systems (including universal health coverage, social protection, and action on determinants), (ii) intensified research and (iii) innovation. To face this scenario, the main strategy includes milestones (for 2020 and 2025) and quantitative targets (for 2030 and 2035) for three high-level indicators: incidence and mortality rates, as well as the percentage of TB patients and their households.^3^ ^,^ ^6^

In 2016, a Brazilian report discussing the Global End TB Strategy program was published as a technical report and a national TB research agenda was proposed to the establishment of the National TB Research Strategy Plan. One of the strategies to address the gap regarding general recommendations was to “create a coordination research group on fundamental and translational research that pursues increased collaboration among various laboratories to better utilise the available knowledge of different groups”.^7^ One of the key endorsed research areas was regarding the investigation on host-pathogen interaction targeting new genetic, molecular, immunological, or metabolic markers, including the use of genotyping and the “omics” supporting epidemiology, new diagnostics methods, studies about new vaccines and more recently new drugs.^7^ ^,^ ^8^

The association between specific *M. tuberculosis* strains and the increase of anti-TB drug resistance is one of the major drivers for mortality rate increase. The investigation of transmission sources and monitoring TB strains by molecular epidemiology studies complemented by molecular typing tools is therefore essential to control TB.^9^

Spacer-oligonucleotide-typing (spoligotyping), mycobacterial interspersed repetitive units - variable number tandem repeat (MIRU-VNTR) typing and the insertion sequence *6110* - restriction fragment length polymorphism (RFLP-IS*6110*) are among the most used genotyping methods for *M. tuberculosis* complex (MTBC) strains. However, due to their different resolving power, only the latter two are used to evaluate TB transmission and perform detailed molecular epidemiology.

MIRU-VNTR is the current reference technique due its higher discriminatory power and reproducibility. Except for RFLP-IS*6110*, because of those characteristics and ease of interpretation and storage, both spoligotypes and MIRU-VNTR based genotypes are stored in large international databases that allow inter-laboratory comparison of patterns while RFLP-IS*6110*, although considerable in number, are mostly composing local databases.^10^ ^,^ ^11^

Through this systematic literature review on the use of RFLP-IS*6110* and MIRU-VNTR, we aimed to (i) describe the Brazilian TB network laboratories structure, (ii) characterise molecular epidemiology studies in Brazil applied to TB; (iii) to study the genetic diversity of *M. tuberculosis* in the country, and (iv) to correlate these data to the national epidemiological scenario of TB.

## MATERIALS AND METHODS

*Data collection of the Brazilian tuberculosis policies* - We have collected the information mainly from the Brazilian National Program of TB Control (NPTC), recently named as General Coordination for the Monitoring of Chronic Conditions Respiratory Transmission Diseases (Coordenação Geral de Vigilância das Doenças de Transmissão Respiratória de Condições Crônicas - CGDR),^12^ which is responsible for establishing guidelines for disease control, manuals, and reports. The national recommendations are updated and disclosed in the technical notes of the NPTC and in the publication of the Brazilian Guidelines for Tuberculosis Control (BGTBC), first edited in 2011, and last published in 2019.^13^

*Data collection on M. tuberculosis genotyping* - Data were collected using PubMed (http://www.ncbi.nlm.nih.gov/pubmed), as well as the Brazilian virtual library BVS (Biblioteca Virtual em Saúde) database. For this, we used the keywords “MIRU-VNTR AND tuberculosis AND Brazil” and “RFLP AND tuberculosis AND Brazil”. All papers were downloaded and information such as data, place and date of samples collections, study period, samples characteristics, genotyping techniques used, the year of publication and principal results obtained) were introduced into a Microsoft Office Excel spreadsheet (Albuquerque, United States).

*Data analysis* - We analysed all papers published until January 28th, 2020 summarised our approach, using the PRISMA flow diagram.^14^

For the present review, for comparison of RFPL-IS*6110-*DNA fingerprints generated in different laboratories and publications, ideally, having access do the DNA patterns together with their respective internal (each lane) or external (each gel) enable us to perform normalisation of the RFLP-IS*6110* patterns appropriate software.^15^ However, we only had access to the figures, either in the format of banding patterns or as digitalised patterns; so, we were restricted to perform a qualitative analysis based on the mean results and on conclusions presented in most of the studies.

For reviewing of MIRU-VNTR patterns on the other hand, for each paper we were able to introduce numeric data into a single Excel file simply by reorganising the order of the published 12, 15 and 24 loci presented. The first 12 MIRU loci positions were composed by: MIRU2 (154), MIRU4 (580), MIRU10 (960), MIRU16 (1644), MIRU20 (2059), MIRU23 (2531), MIRU24 (2687), MIRU26 (2996), MIRU27 (3007), MIRU31 (3192), MIRU39 (4348) and MIRU40 (802) was adopted. For the next 12 VNTRs of the 24-MIRU-VNTR patterns, we organised according to the ETR, MTUB and QUB scheme, being ETR-A (2165), ETR-B (2461), ETR-C (577), MTUB 04 (424), MTUB 21 (1955), MTUB 29 (2347), MTUB 30 (2401), MTUB 34 (3171), MTUB 39 (3690), QUB 11 (2163b), QUB 26 (4052) and QUB 4156 (4156). For 15 MIRU-VNTR typing, the same order was adopted but removing nine loci: MIRU2 (154), MIRU20 (2059), MIRU23 (2531), MIRU24 (2687), MIRU27 (3007), MIRU39 (4348), ETR-B (2461), MTUB 04 (424) and MTUB 29 (2347).

Recent transmission was estimated by the N-1 method, using the mathematical model: number of clustered isolates minus (-) number of clusters divided (/) by the total number of isolates.^16^ The allelic diversity (*h*) at MIRU-VNTR loci was calculated according to Hunter-Gaston index^17^ using Bionumerics (Applied Maths, Sint-Martens-Latem, Belgium) for each set of 12, 15 and 24 loci and defined as “highly discriminant” (*h* > 0.6), “moderately discriminant” (0.3 ≤ *h* ≤ 0.6) or “poorly discriminant” (*h* < 0.3).

In addition, we have used TBminer (https://info-demo.lirmm.fr/tbminer/)^18^ to predict the MTBC lineages from MIRU-VNTR profile.

*Disease distribution based on geographic mapping* - The boundaries of the regional divisions of Brazil (States and Regions) applied presently were obtained on the website of the Brazilian Institute of Geography and Statistics (IBGE) (https://www.ibge.gov.br/).^19^ The coordinates of the institutions were obtained from Google Maps (https://www.google.com.br/maps). Data processing, interpretation, visualisation, and spatial analysis were performed via ArcGIS software (http://www.arcgis.com/). TB incidence was classified into five levels according to the WHO and being either absence of: no cases (white colour), low (1-10 - green), medium (11-50 - yellow), high (51-100 cases - orange) and very high number (> 100 - red) cases per 100,000 hab.

## RESULTS

*The Brazilian organisational structure for TB policies* - The NPTC is linked to three governmental spheres and coordinated by the so-called Unified Health System (UHS) (Sistema Único de Saúde - SUS) that legally establishes administrative competence at the federal, state, and municipal level. These spheres are composed of the Ministry of Health, the State Health Secretariats (one for each of the 26 states and the Federal District) and the Municipal Health Secretariats, each having their respective technical and administrative sectors.^13^

**Fig. 1:**
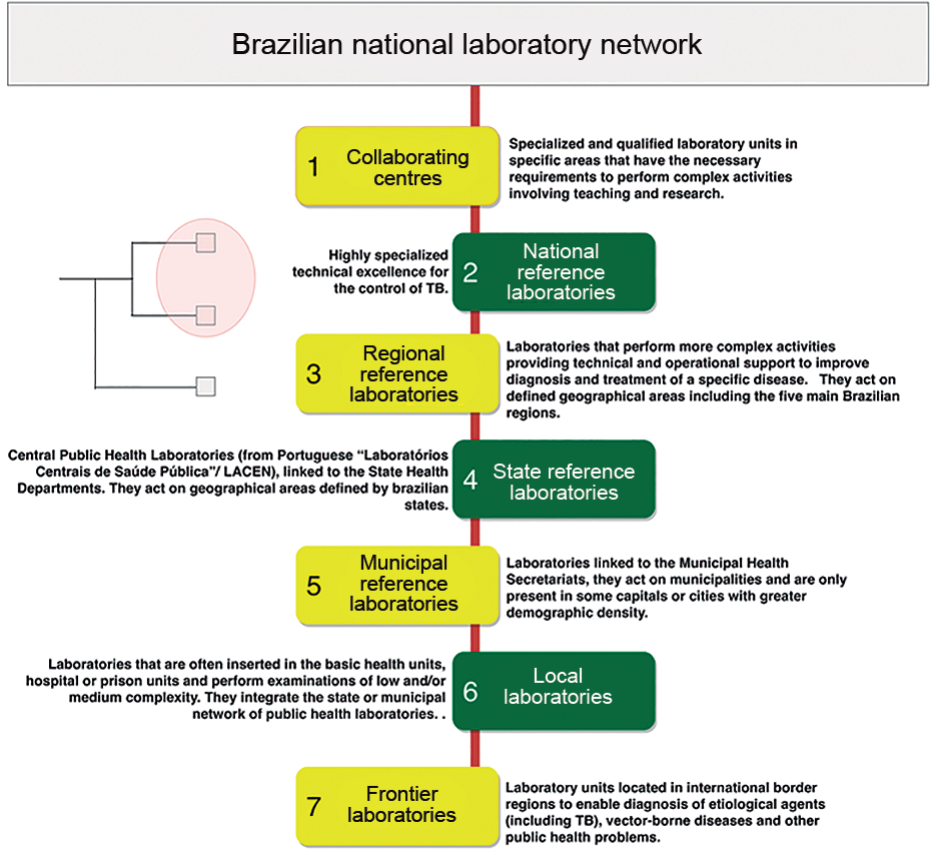
the Brazilian National System of Public Health Laboratories network classified by degree of complexity highlighting the two levels capable to elaborate a national genetic database for tuberculosis (TB) surveillance.

The National System of Public Health Laboratories (Sistema Nacional de Laboratórios de Saúde Pública - SISLAB) consists of a national network of laboratories, organised in sub-networks in a hierarchical way and with different degrees of complexities of activities related to health surveillance. There are seven laboratory categories^13^ as represented in Fig. 1.

The Brazilian Guidelines focus basically on clinical recommendations regarding the standardisation of case finding and treatment actions with little, or no information related to genotyping data generated in Brazilian studies.

For routine TB diagnosis in clinical specimens, besides chest X-ray, collection of sputum samples for acid-fast bacilli staining (Ziehl-Neelsen and/or auramine-rodamine stain) are culture in solid (Lowenstein-Jensen or Ogawa-Kudoh) (AFB) or liquid media (BACTEC MGIT 960) are performed. However, between 2014 and 2015, the Brazilian NPTBC implemented the molecular diagnostics technology Xpert^®^ MTB/RIF (rifampicin) in 92 municipalities with high disease burden.^20^ More recently, the identification of MTBC isolates by the rapid immunochromatographic test SD-Bioline TB Ag MPT 64 (Standard Diagnostics, Seoul, South Korea) was implement in Brazil.

The phenotypic drug-susceptibility tests (DST) for first line drugs are performed in all State Reference Laboratories named Laboratório Central (LACEN) and are based on the MGIT-960 SIRE kit (MGIT-960; Becton Dickinson Diagnostic Systems, Sparks, MD). At municipality level, the molecular drug-susceptibility test Xpert-Ultra^®^ MTB/RIF (Cepheid, Sunnyvale, EUA)^21^ is performed for detection of RIF-R while at the regional reference laboratories, GenoType^®^MTBDR*plus* and GenoType^®^MTBDR*sl* (Hain Lifescience GmbH, Nehren, Germany) are used, detecting respectively mutations associated with rifampicin and isoniazid resistance, and mutations associated with fluoroquinolones and second-line injectable drugs.

Currently, the DST for second-line drugs is carried out only in three laboratories in Brazil: at the National Reference Centre (Centro de Referência Professor Hélio Fraga - CRPHF) and at the Laboratório de Bacteriologia e Bioensaios both belonging to the Oswaldo Cruz Foundation (Fundação Oswaldo Cruz - Fiocruz) in Rio de Janeiro, and at the LACEN in São Paulo.

Focusing more on epidemiological surveillance, the Notifiable Diseases Information System for Tuberculosis (SINAN-TB) is the main source for professionals of health surveillance services for data analysis and for planning and monitoring actions towards TB control at the three government levels: federal, state and municipality. A recent study demonstrated the current algorithm used by SINAN-TB, which has a unique identifier per person, integrated with other information systems and built on new technologies, so that TB data transfer and analysis is more streamlined in Brazil. Interestingly, the only results from a molecular diagnostic test that are included in the SINAN-TB are those of the Xpert MTB/RIF assay.^22^

**Fig. 2:**
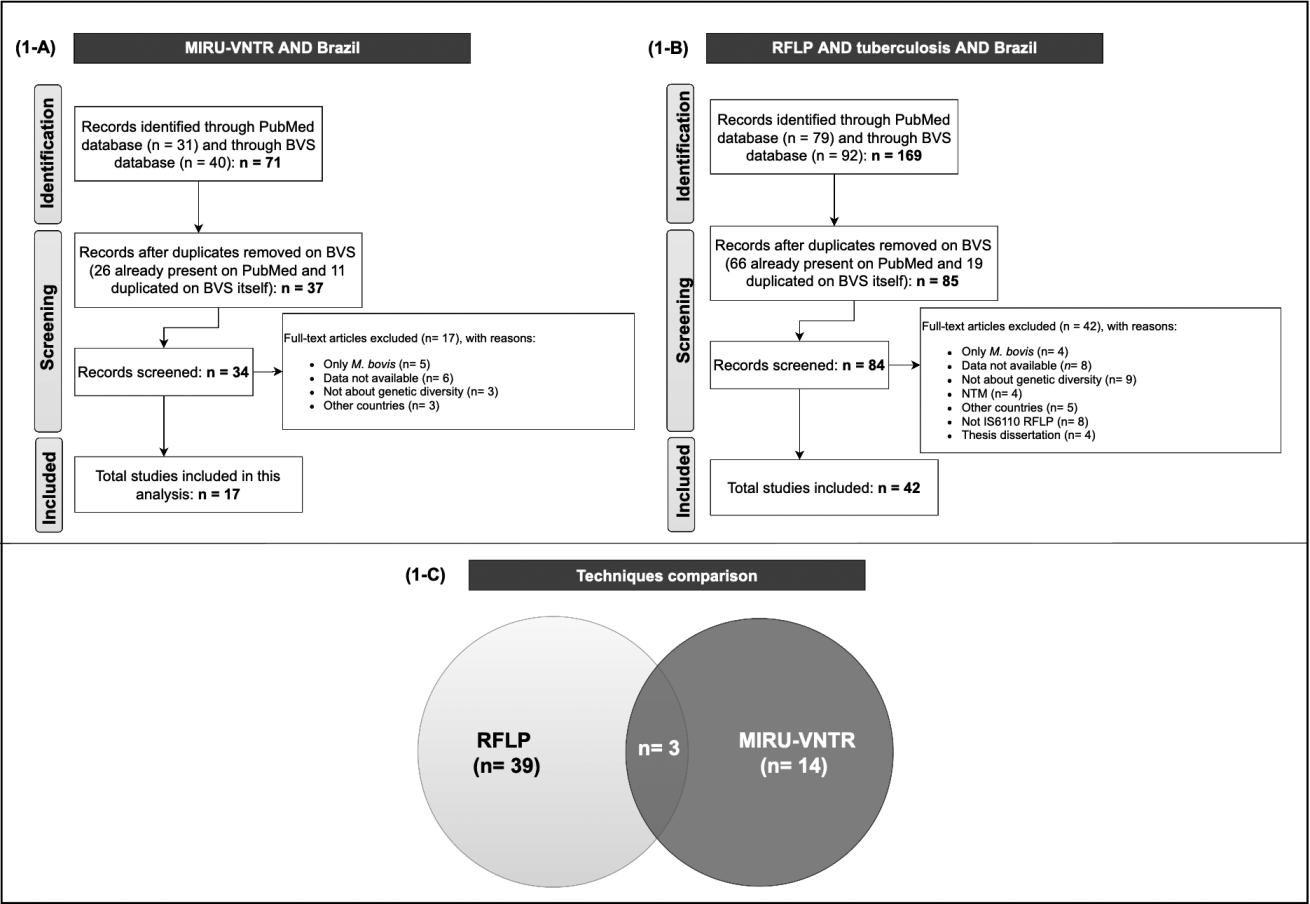
the PRISMA flow diagram for each genotyping technique demonstrating the total of studies selected for this literature review: 1-A) mycobacterial interspersed repetitive unit-variable variable number tandem repeat (MIRU-VNTR) and 1-B) restriction fragment length polymorphism (RFLP-IS*6110*). 1-C) The 57 studies of *Mycobacterium tuberculosis* genotyping in Brazil and their distribution according to each method.

*Data analysed* - Among a total of 240 manuscripts published between 1996 and 2019, 169 on RFLP-IS*6110* and 71 on MIRU-VNTR, we considered 56 eligible for our study. The BVS database constitutes mostly of PubMed publications along with some duplicated articles within the rest of its own databases. Fig. 2 demonstrates details about the screening process and finally: 17 manuscripts on MIRU-VNTR 17^23^ ^-^ ^39^ and 42 on RFLP-IS*6110* were considered;^23^ ^,^ ^26^ ^,^ ^31^ ^,^ ^32^ ^,^ ^40^ ^-^ ^75^ three manuscripts considered both methodologies.

In the case of MIRU-VNTR data, some articles were excluded because they only contained information on *M. tuberculosis* var. *bovis* (n = 5);^76^ ^,^ ^77^ ^,^ ^78^ ^,^ ^79^ ^,^ ^80^ no data on genotyping were available (n = 6);^49^ ^,^ ^66^ ^,^ ^81^ ^,^ ^82^ ^,^ ^83^ ^,^ ^84^ incomplete information on genetic diversity (n = 3)^85^ ^,^ ^86^ ^,^ ^87^ or genotyping data is mixed with samples from other countries (n = 3).^84^ ^,^ ^88^ ^,^ ^89^

Regarding manuscripts on RFLP-IS*6110*, we excluded those with data on *M. bovis* only (n = 4);^90^ ^,^ ^91^ ^,^ ^92^ ^,^ ^93^ without visible on genotyping (n = 8)^33^ ^,^ ^94^ ^,^ ^95^ ^,^ ^96^ did not present data regarding genetic diversity (n = 9);^24^ ^,^ ^97^ ^,^ ^98^ ^,^ ^99^ ^,^ ^100^ ^,^ ^101^ ^,^ ^102^ ^,^ ^103^ were related to nontuberculous mycobacteria (NTM) (n = 4);^104^ ^,^ ^105^ ^,^ ^106^ ^,^ ^107^ were performed in other countries (n = 5);^88^ ^,^ ^108^ ^,^ ^109^ ^,^ ^110^ ^,^ ^111^ did not target IS*6110* for RFLP (n = 7)^112^ ^-^ ^118^ or were data presented as part of a thesis manuscript and had not been peer reviewed (n = 4).

We observed that RFLP-IS*6110* analysis was the first technique to evaluate genetic diversity of *M. tuberculosis* in Brazil over two decades ago, and data using this technique are still being published. This technique has nowadays been substituted almost completely by MIRU-VNTR typing (Supplementary data I).

**Fig. 3:**
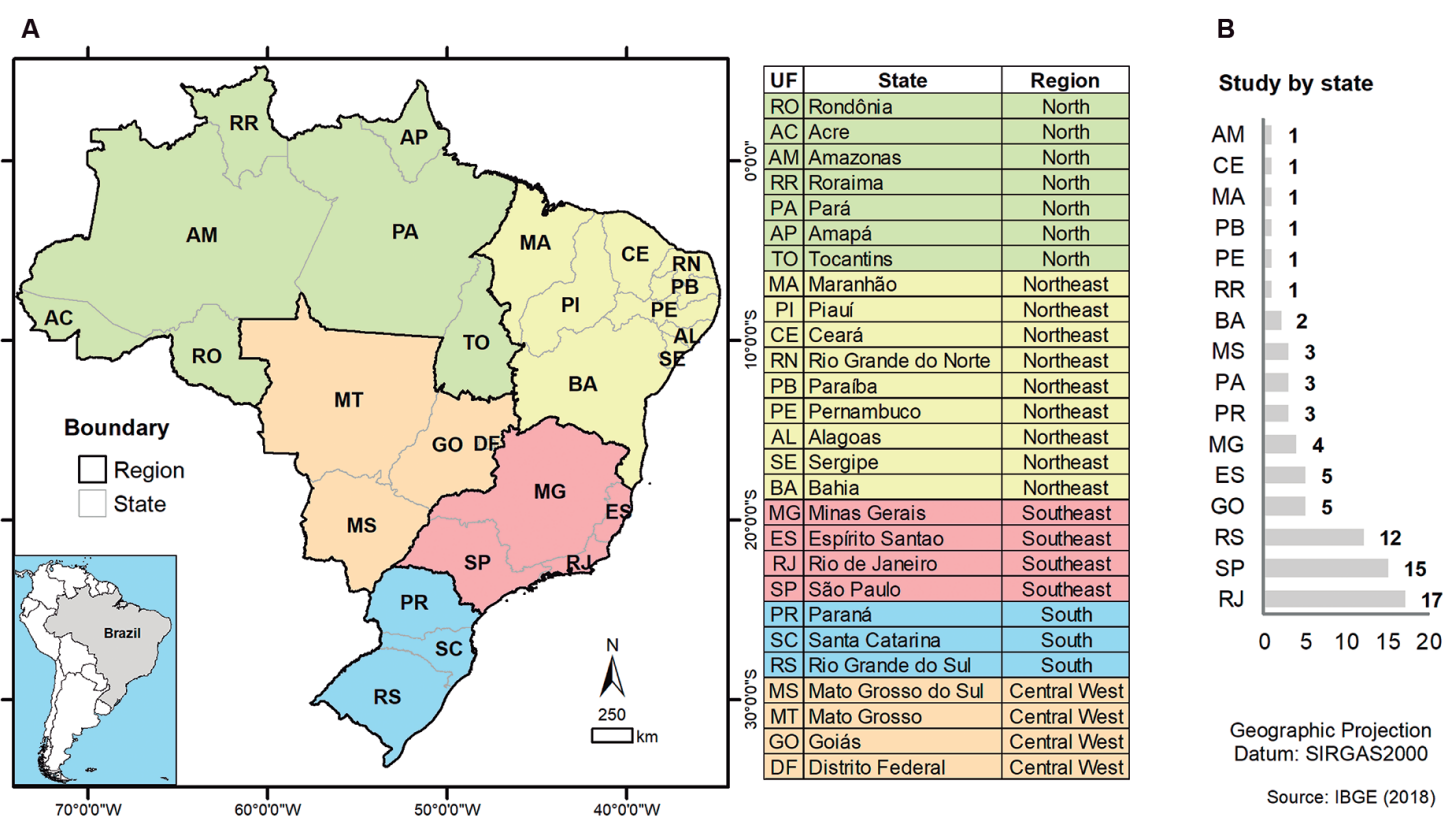
studies distribution based on genotyping by restriction fragment length polymorphism (RFLP-IS*6110*) and mycobacterial interspersed repetitive unit-variable variable number tandem repeat (MIRU-VNTR) in Brazil. (A) Spatial localisation; (B) number of studies by states.

The geographical map of Brazil with TB incidence, study distribution based on sampling and manuscript authorship is presented by Fig. 3. Fig. 3A demonstrates the spatial location of the country divided in five regions and 27 states demonstrating a considerable difference of incidence per state, with the states of Amazonas (AM - North) and Rio de Janeiro (RJ - Southeast) presenting the highest values (72.9 and 66.3 per 100.000 inhabitants).

Studies using MIRU-VNTR or RFLP-IS*6110* were performed in all regions of Brazil and in 16 (59%) of the states (including the Federal District) but most (n = 40/56) were performed in the Southeast region, including 17 in Rio de Janeiro and 15 in São Paulo states; and in the South, represented by 12 studies from Rio Grande do Sul and three from Paraná states. In the North, three studies were represented from Pará State while the Central West region harbored five studies, all from Goiás State (Fig. 3).

*RLFP data-analysis* - Among the Brazilian regions, the largest number of studies, using RFLP-IS*6110*, was observed in the Southeast region, with more than 70% of all publications, followed by the South and Central West regions that, together, do not reach 25%. The description of clustering rate and number of IS*6110* copies can be found in Table I, and the Southeast region includes studies comprising all the constituent states with a clustering in the general population ranging from 6 to 56.2%. Among the vulnerable populations studied, transmission rate was observed of 53% among HIV patients (71) and 84% among prisoners.^52^

**TABLE I:** Summary of restriction fragment length polymorphism-IS*6110* (RFLP-IS*6110*) genotyping publications

	States	No. of studies^*a*^	%	% by region	No. of IS*6110*	% in cluster
Southeast	SP	11	28.2	74.4	2 to 21	22 to 56
	ES	5	12.5		NR	40 to 48
	RJ	11	28.2		2 to 22	19 to 84^*b*^
	MG	2	5.1		1 to 18	6 to 25
South	PR	0	0	10.3	-	-
	SC	0	0		-	-
	RS	4	10.3		1 to 18	36 to 69^*c*^
Central West	GO	2	5.1	10.3	1 to 14	0
	MT	0	0		-	-
	MS	2	5.1		4 to 17	64 to 69^*d*^
	DF	0	0		-	-
North	TO	0	0	2.6	-	-
	PA	0	0		-	-
	AP	0	0		-	-
	RR	1	2.6		NR	30
	AM	0	0		-	-
	RO	0	0		-	-
	AC	0	0		-	-
Northeast	MA	0	0	2.6	-	-
	PI	0	0		-	-
	CE	0	0		-	-
	RN	0	0		-	-
	PB	0	0		-	-
	PE	0	0		-	-
	AL	0	0		-	-
	SE	0	0		-	-
	BA	1	2.6		2 to 16	27
TOTAL		39	100	100	1 to 22	0 to 84

*a*: for these calculations, articles that analysed samples from more than one state in the same study were disregarded; *b*: study with inmate population; *c*: tuberculosis multidrug resistant (TB-MDR) population study; *d*: study with indigenous population; NR: not reported.

For São Paulo, the state with the biggest population and economic growth, some specific populations showed high clustering rate, such as patients with resistant TB (52.3%),^46^ extensively resistant (52.8%)^54^ and prisoners (55.9%).^60^ Rio de Janeiro and São Paulo were the municipalities with the highest number of publications using RFLP-IS*6110* (11 each) and demonstrated an increase in the rate of grouping/transmission over time. Among the studies analysed, one study conducted in the central region of São Paulo reported the highest clustering rate (56.2%) and in this study, they sought to verify the impact of migration on the recent transmission of TB. Despite the high percentage found in that study, there seems to be limited contribution of migration in the transmission of TB to Brazilians and vice versa.^50^

For Espírito Santo State, the clustering rate ranged from 40 to 48%; however, as most studies comprised periods of time and/or were overlapping, it was not possible to infer a tendency for increase or decrease in this particular state. However, the incidence of TB in Espirito Santo state seems to be highly influenced by a small set of strains that circulate actively.^53^ ^,^ ^69^

Data from Minas Gerais State shows that its rate of clustering was the lowest in the Southeast region, ranging between 6.4% and 25.4%, suggesting a low recent rate of TB transmission in this state, including patients with MDR-TB; this might have been influenced however by the low sampling.^26^

Five publications were from the South region, all from the state of Rio Grande do Sul; strains with one to 18 copies of IS*6110* have been reported and the percentage of grouping ranged from 36 to 42.9% for the general population and from 38% to 68.6% for the population with MDR-TB. Despite the small number of publications, studies with MDR-TB patients suggesting an increase of MDR-TB transmission during the studied period, a particular problem of this Southern state and probably associated with HIV infection.

In Mid-West Brazil, more specifically in the Goiás State, polymorphism is observed in TB resistant and TB-MDR strains, suggesting a high rate of primary resistance. Two studies in Mato Grosso State, carried out exclusively upon the indigenous population, demonstrated a high clustering rate of 63.5%^64^ and 69%^51^ typical for high transmission rates among such populations.

In the Northeast region of the country, the only study we found was that of Silva et al.^119^ (evaluating isolates from Bahia State that had been collected between March and June 2008, reporting *M. tuberculosis* with a number of IS*6110* copies ranging between 2 and 16, with a cluster rate of 26.7%.

Similarly, the North region was also represented by a single study conducted in the State of Roraima which borders with Venezuela and Guyana and has an important portion of their TB cases associated with indigenous population, constituting 70% of TB cases (2015-2016) and presenting a clustering rate of 30%.^31^ This clustering rate is low when compared to other regions of the country what might be related to a large flow of people, being a border region.

**Fig. 4:**
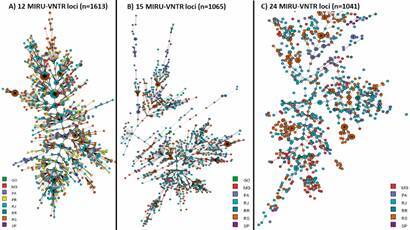
minimum spanning trees (MST) demonstrating the genetic diversity of *Mycobacterium tuberculosis* in Brazil based on consideration of 12, 15 or 24 mycobacterial interspersed repetitive unit-variable variable number tandem repeat (MIRU-VNTR) alleles and considering a different dataset according to the method’s sampling. Samples are coloured according to state origin: Goiás (GO), Minas Gerais (MG), Rio de Janeiro (RJ), Rio Grande do Sul (RS), São Paulo (SP), and Pará (PA).

*MIRU-VNTR data-analysis* - We obtained MIRU-VNTR genotypes from 1,613 MTBC isolates and conducted the analysis using 12, 15 or 24 MIRU-VNTR alleles for constructing genetic patterns. Patterns of 24-MIRU-VNTR were available for 1,041 (64.5%) isolates from all states except from Goiás and Paraná. The data demonstrated that genotypes are not exclusively of a specific state (Fig. 4). Because 24-MIRU-VNTR typing is also considered adequate for phylogenetic analysis, we are puzzled by the bimodal and mostly region-independent grouping within the MST tree with strains from Rio de Janeiro State at the central node (Fig. 4C).

Upon analysis of the number of studies on MIRU-VNTR typing in the country, we observed that the Southeast and South regions present the highest number (82.4%; 14/17) and in general, these studies demonstrated that the MIRU-VNTR present a high discriminatory power. Two of these with the largest sampling^28^ ^,^ ^29^ ^,^ ^30^ showed a low rate of clustering in the *M. tuberculosis* population (0.13 and 0.28, respectively). Additionally, two studies^32^ ^,^ ^33^ investigated isolates from the same patient with up to three different loci and upon further characterisation, such closely related MIRU-VNTR types, demonstrated to belong to the same strain.

The clustering rate according to each MIRU-VNTR set of 12, 15 and 24 loci was 73%, 33% and 28%, respectively (Table II). Among the 24 and 15 loci evaluated, 4052_QUB26, 2163b_QUB11b, 424_Mtub04, 802_MIRU40, 1955_Mtub21, 2696_MIRU26, were considered highly discriminant. Regarding the 24 and 15 loci evaluated, 802_MIRU40, 2696_MIRU26, 2531_MIRU23, were considered highly discriminant and present in 12 and 24 loci analysis, while 802_MIRU40, 2696_MIRU26 and 960_MIRU10 were highly discriminant and commonly present in 12 and 15 loci analysis.

**TABLE II:** Clustering analysis of mycobacterial interspersed repetitive units - variable number tandem repeat (MIRU-VNTR)

Typing methods	n	No. of different patterns	No. of clusters	No. of clusters isolates	No. of unique isolates	% in cluster	Size of clusters
MIRU-VNTR 12 loci	1,613	26	11	40	15	73%	2-7
MIRU-VNTR 15 loci	1,065	821	106	350	715	33%	2-23
MIRU-VNTR 24 loci	1,041	845	91	287	754	28%	2-11

The 802_MIRU40 and 2696_MIRU26 were the highly discriminant among the three dataset and 960_MIRU10 were highly discriminant in 12 and 15 loci and moderately discriminant in 24 loci analysis (Supplementary data II).

The assignation (lineage and/or sublineage) of the strains using TBminer is presented in Supplementary data III. All assignations in green are reliable (at least 2 classifications providing the same result). Lineage 4, mainly Latin-American Mediterranean (LAM) is predominant in all states, but Lineage 1 is mainly isolating from patients in the Pará State, and Lineage 3 is predominantly from Rio Grande do Sul State. Although the observation of the potential concentration *M. bovis* in Goiás, and Lineage 5 (*M. africanum*) in Rio de Janeiro states, there is not a consensus between the two classifications available.

## DISCUSSION

This study presents the data on the genetic diversity of *M. tuberculosis* and TB transmission within the new era of global TB monitoring, discussing aspects of TB molecular epidemiology in Brazil previously pointed out in a translational research perspective - “from bench to bedside”.^120^

In the light of the Genomic Era, a recent study conducted in England described benefits of TB molecular strain-based cluster investigations (CIs) into a translational approach by identifying new epidemiological links between cases and taking public health action, as well as refuting transmission and saving resources.^121^ According to these results, molecular typing is efficient for decreasing transmission and adds value for improving public health in low disease prevalence and high resource setting.

Even though Xpert^®^ MTB/RIF has been implemented in Brazil since 2014, this test does not provide information about MTBC lineages or transmission that could be useful for epidemiological studies and clinical decision-making. Current trends in this direction, point to the use of a new technologies that are able to provide both molecular DST and epidemiological information based on next generation sequencing (NGS) using whole-genome sequencing (WGS).^122^

A recent review^6^ showed that between 2009 and 2016, a total of $4.6 billion was invested into TB research, mostly for the development of new diagnostics tools, drugs, and vaccines (61%). Studies on genetic variability are welcome but should go a step further towards translational sciences. Therefore, genotyping tools are important not only to achieve a faster diagnostic and treatment scheme, but also to be implemented at least at the level of regional reference laboratories as a measure of monitoring and controlling TB.

The Brazilian TB surveillance actions include home visit for new case and summoning of possible cases of TB infection in hospitals and other institutions, as well as follow-up and closure of cases.^13^ ^,^ ^22^ Currently, strain typing (that would be preferably by 24-loci MIRU-VNTR typing) is not part of routine surveillance in any institution in Brazil, only for basic research.

The present study demonstrates that over the last two decades, MIRU-VNTR has been used less for genotyping than the previous gold standard technique (RFLP-IS*6110*), not only because of earlier implementation of the latter (1993 *versus* 2008), but also due to its higher cost. This is of particular importance for a country with a considerable TB incidence such as Brazil (rate of 33.5 per 100,000 inhabitants). However, implementation of such technology to screen only MDR-TB cases would be already a great step ahead.

The use of international databases not only allows local or national genotyping studies, but evaluation of genetic composition of *M. tuberculosis* strains on a larger and even on a global level. In particular for MIRU-VNTR, besides SITIVIT2, there is another international database that allows comparison and classification to the lineage level of local genotypes (MIRU-VNTR*plus* - http://www.miru-vntrplus.org), which has a collection of 186 strains representing the major MTBC lineages. For each strain species, lineage and epidemiologic information is stored together with information regarding the copy numbers of 24 MIRU loci, spoligotyping patterns, regions of difference (RD) profiles, single nucleotide polymorphisms (SNPs), susceptibility data and RFLP-IS*6110* fingerprint images for all isolates.^123^ ^,^ ^124^ However, because this database is limited to the input of genotypes from 500 isolates per analysis and our sampling was composed of 1,613 entries for MIRU-VNTR, we used the Bionumerics v.7.6 software (Applied Maths, Sint-Martens-Latem, Belgium) for analysis.

The limitations of RFLP-IS*6110* are due to the requirement of large amounts of purified DNA in a more complicated methodology with extensive and laborious steps during data analysis for comparison of data generated in different laboratories by considering internal and external molecular weight markers. Comparison of such data requires the use of specialised programs such as Bionumerics and considerable experience on part of the user, for pattern analysis.

Although a considerable number of studies in Brazil performed genotyping by RFLP-IS*6110* have been published*,* they mostly report on regional patients where a single laboratory analysed the samples without inter-laboratory comparison because of the afore mentioned characteristics of the technique. In addition, no robust international database of RFLP-IS*6110* profiles is accessible. There is one centralised database at the National Institute for Public Health and the Environment (RIVM, Bilthoven, the Netherlands) but only accessible for collaborators.^73^ ^,^ ^94^

Although this technique has been used to date, MIRU-VNTR analysis has already proved its robustness and equivalence to the results obtained by RFLP- IS*6110*.^125^ Moreover, it still has the advantage of allowing the analysis of isolates with fewer copies of IS*6110* which is not considered a good RFLP-IS*6110* method for these cases.

The higher discriminatory power of MIRU-VNTR compared to other genotyping techniques is already widely known,^126^ also showing a range of polymorphism, such that loci 10, 23, 26, 31 and 40 have greater discriminatory power than the others. Additionally, its value has been demonstrated for detecting relapse cases, reinfection, and mixed infections.^127^

Comparing the MIRU-VNTR discriminatory power, this study corroborates a recent review evaluating 56 studies (39 from Asia, seven from America, six from Africa, three from Europe and one from a different country),^128^ demonstrated that MIRU10, MIRU26, QUB26, MIRU40, QUB11b and Mtub21 was reported to be the loci with the highest discriminatory powers (*h* > 0.6), in Brazilian population, we also present MIRU 23 and Mtub04 with high discriminatory power. These eight loci can be considered in studies that need to be faster and less costly. Studies supported by the Brazilian government exploring and describing the MTBC genetic diversity into the five main regions have correlated the emergence of drug resistant-TB to RD^rio^ (LAM sublineage) strains in South and Southeast regions^23^ ^,^ ^129^ and to the T lineage in the North.^130^

Concerning the phylogenetic network, the central position is not meaningful in the context of many diverse isolates. The ancestor that gave rise to all these strains has no good representative today, in this way the centre is highly dependent on the frequency of the samples, and the strains that have by chance quite average values for the genotyped loci. There is little spatial structure in Brazil. This can also be seen in the assignation to lineages as described above. Regarding the lineages, the Brazilian profile observed in this demonstrate the higher frequency of Lineage 4, mainly LAM genotype and the higher frequency of Lineage 1 in Pará State.^130^ ^,^ ^131^ ^,^ ^132^ ^,^ ^133^ The presence of *M. tuberculosis* var. *bovis* among human strains was not reported so far, and *M. tuberculosis* var. *africanum* was recently reported as a single isolate from a patient from Pará State.^134^

This study has some limitations, and they are mostly related to data correlation since some articles do not show genotype data (the number of each MIRU-VNTR loci, or RFLP-IS*6110* profile), so they were excluded from the analysis. Some publications repeat data from previous studies without allowing sample identification, so it was not possible to evaluate the real frequency of genotypes isolates per Brazilian state or region.

On December 23rd of 2019, the Brazilian Secretary of Health Surveillance has published the list of approved National and Regional Reference Laboratories for TB and atypical mycobacteria (NTM), aiming at the establishment of the National Network of Public Health Laboratories for the next 5 years. Institutes on the national level are the National Reference Laboratory Professor Hélio Fraga (CRPHF) of the Fundação Oswaldo Cruz. At the regional level are the Regional Reference Laboratories: The Laboratory of Bacteriology and Bioassays of the National Institute of Infectious Diseases Evandro Chagas (INI, FIOCRUZ), the Central Public Health Laboratory of Amazonas (LACEN, AM), the Central Laboratory of Public Health of Espírito Santo (LACEN, ES); and the Central Public Health Laboratory of the Federal District (LACEN, DF).

Regarding the advances on technology evolution, studies have demonstrated that WGS has the greater discriminatory power for epidemiological compared to genotyping methods. For example, to track TB transmission, it was already established that, based on WGS data, a genetic distance from zero to five SNPs separating patient isolates, are present in linked cases such as household contacts; a genetic distance from five to 12 SNPs is for related cases and more than 12 SNPs was defined to classify epidemiologically unrelated cases.^135^ ^,^ ^136^ Besides that, compared to the commercial genotyping methods or Sanger sequencing, analysis based on WGS display a greater panel of mutations associated to drug resistance.

Taking out 23 *M. bovis* genomes, there are few studies in Brazil related to WGS applied to *M. tuberculosis* so far (around 765 genomes): five related to drug resistance characterisation^122^ ^,^ ^137^ ^,^ ^138^ ^,^ ^139^ ^,^ ^140^ and four related to epidemiological approach^29^ ^,^ ^141^ ^,^ ^142^ ^,^ ^143^ we did not include them in this current analysis. Such national studies confirm the potential of WGS for molecular epidemiology approach compared to genotyping. In Supplementary data IV there is a list of all published MTBC genomes isolated in Brazil so far, which is the first version of what should become an interactive database of *Mycobacterium* genomes from patients from Brazil, including MTBC, *M. leprae* and NTM presently under construction at http://www.ioc.fiocruz.br/gemibra/. Part of these MTBC genomes are also available at a website http://cplp-tb.ff.ulisboa.pt/, a TB Molecular Epidemiology Database for the Community of Portuguese Speaking Countries (CPLP).^89^

Even through the natural progression towards WGS is going on, applying MIRU-VNTR and creating a national genotyping database for TB surveillance is more feasible, at least for a while, than WGS at the regional and national reference laboratories. However, in parallel, we could give rise to an interactive national database for WGS, focusing on the genetic structure of MTBC in Brazil, for research and for TB surveillance.

Thus, a similar long-term analysis performed in this study could address a better understanding of the TB dynamics in all of Brazil and refocus the attention towards the gold standard of surveillance. This is the same direction that Singapore has taken by demonstrating that there is a large and heterogeneous distribution of MTBC strains. A universal MTBC typing program coupled with enhanced contact investigations may be useful in further understanding the transmission dynamics of TB locally.^144^

In conclusion

Tracing TB cases and their contacts is of vital importance for the control of TB in high burden countries like Brazil. Research on TB genetic diversity and molecular epidemiology in Brazilian territory was more frequent in South and Southeast and it is imperative to reinforce the need of molecular epidemiology surveillance in the central and northern states. This could be achieved by the intensive training of more laboratory professionals and supply of the materials needed to perform the technique. A high but heterogeneous rate of TB transmission was observed in Brazilian regions. This study highlights the importance of including genotypic analysis by MIRU-VNTR in TB surveillance, at least of drug-resistant cases, and of maintaining a hierarchical flow of data between laboratories in the NPCT network. Thus, we propose an implementation of molecular typing techniques for TB transmission detection based initially on MIRU-VNTR towards WGS, as well as the creation of a national database would improve our efforts to decrease the incidence of this challenging disease.

List of abbreviations

AC: Acre; AFB: Acid-fast bacilli; AL: Alagoas; AM: Amazonas; AP: Amapá; BVS: Biblioteca Virtual em Saúde; BA: Bahia; CE: Ceará; DF: Distrito Federal; DR: direct repeat; ES: Espírito Santo; GIS: geographic information system; GO: Goiás; HBCs: high burden countries; HIV: human immunodeficiency virus; LAM: Latin-American; MA: Maranhão; MDR: multidrug resistant; MG: Minas Gerais; MIRU-VNTRs: mycobacterial interspersed repetitive units-variable tandem repeats of DNA tandem repeats; MS: Mato Grosso do Sul; MT: Mato Grosso; MTBC: *Mycobacterium tuberculosis* complex; MST: minimum spanning tree; PA: Pará; PB: Paraíba; PE: Pernambuco; PI: Piauí; PR: Paraná; RFLP: restriction fragment length polymorphism; RJ: Rio de Janeiro; RN: Rio Grande do Norte; RO: Rondônia; RR: Roraima; RS: Rio Grande do Sul; SC: Santa Catarina; SE: Sergipe; SDG: sustainable development goals; SISLAB: Sistema Nacional de Laboratórios de Saúde Pública; SP: São Paulo; SUS: Sistema Único de Saúde; TB: Tuberculosis; TO: Tocantins; UHS: unified health system; WHO: World Health Organization; XDR: extensive drug resistant.
