# Supplementary material for: Molecular epidemiology of Mycobacterium tuberculosis in Brazil before the whole genome sequencing era: a literature review
Source: Mem Inst Oswaldo Cruz. 2021 Mar 15;116:e200517. doi: 10.1590/0074-02760200517 (PMC7976556; doi:10.1590/0074-02760200517)
Supplement: Supplementary file 4 [file 1678-8060-mioc-116-e200517-s4.pdf]

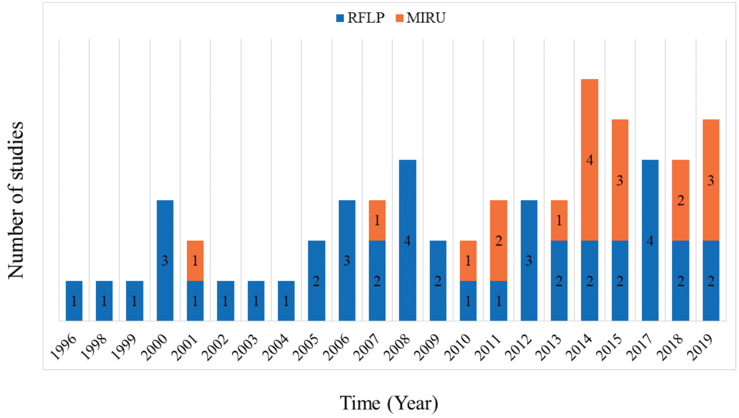

Molecular epidemiology studies reporting genotyping of *Mycobacterium tuberculosis* in Brazil from 1996 to 2019.

CC BY
